# Transforming uncertainty into exploration: effects of text-to-image AI on EFL learners' writing anxiety, motivation, and performance

**DOI:** 10.3389/fpsyg.2026.1866688

**Published:** 2026-08-24

**Authors:** Dan Chen

**Affiliations:** 1 School of International Education, Ningxia Medical University, Ningxia, China; 2 Department of English, Faculty of Modern Languages and Communication, Universiti Putra Malaysia, Selangor Darul Ehsan, Malaysia

**Keywords:** anxiety, EFL writing, generative AI, motivation, text-to-image, uncertainty

## Abstract

**Introduction:**

English as a Foreign Language (EFL) writing is inherently fraught with linguistic uncertainty, which frequently triggers anxiety and stifles learner motivation. While generative AI (GenAI) is increasingly adopted, current research predominantly treats these tools as text-generation shortcuts for final writing products, leaving the cognitive and affective mechanisms of multimodal GenAI as a process-oriented scaffold largely underexplored.

**Method:**

To bridge this gap, this study investigated the effects of a text-to-image (T2I) AI-supported visual narrative intervention on 78 EFL undergraduates' writing anxiety, motivation, and performance through an 8-week quasi-experiment. Specifically, the intervention required the experimental group to engage in an iterative cycle of text input, image generation, and prompt refinement to construct their drafts, whereas the control group employed traditional text-only drafting.

**Results and discussion:**

Quantitative results revealed that the experimental group exhibited significantly lower writing anxiety, alongside enhanced situational writing motivation and a modest but significant improvement in writing performance, compared to the control group. Qualitative thematic analysis of retrospective interviews indicated that, during the intervention, the T2I AI transformed linguistic uncertainty from a psychological threat into a gamified exploration, although the durability of this shift beyond the intervention period remains to be established. The immediate, non-judgmental visual feedback functioned as an autonomy-supportive scaffold, driving learners to notice the gap and autonomously engage in lexical risk-taking and syntactic refinement. However, as the intervention bundled visual feedback with AI immediacy and interactivity, the specific contribution of the visual modality warrants further investigation. This study contributes to reframing the understanding of uncertainty in GenAI-supported EFL writing and highlights the pedagogical affordances of leveraging multimodal AI as a collaborative, affective scaffold rather than a mere linguistic shortcut.

## 1 Introduction

Writing in English as a Foreign Language (EFL) is an inherently complex cognitive activity marked by substantial linguistic and rhetorical uncertainty (Hyland and Milton, 1997). Learners must continuously make decisions about lexical choice, syntactic accuracy, and pragmatic appropriateness, often under conditions of limited linguistic confidence. When faced with such uncertainty, many students experience a heightened sense of vulnerability, particularly during the initial stages of writing. This perceived lack of control frequently gives rise to foreign language writing anxiety (FLWA), a well-established affective barrier that includes cognitive anxiety, somatic anxiety, and avoidance behavior (Cheng, 2004; Horwitz et al., 1986). When uncertainty is experienced primarily as a threat, it can undermine intrinsic motivation, overload working memory, and ultimately impair writing performance (MacIntyre and Gardner, 1991; Rahimi and Zhang, 2019; Sabti et al., 2019; Tahmouresi and Papi, 2021). Addressing this affective bottleneck is therefore essential for cultivating resilient and motivated learners and for advancing broader educational goals related to learner wellbeing.

At the same time, emerging perspectives in educational psychology and neuroscience suggest that uncertainty should not be viewed solely as a deficit to be eliminated. As (Dayan and Jyu 2003, p. 171) observe, “behavioral learning has uncertainty at its heart.” When appropriately scaffolded, uncertainty can stimulate attention, activate adaptive cognitive processes, and encourage exploratory learning (Giustiniani et al., 2020; Soltani and Izquierdo, 2019). In contrast, many conventional EFL writing interventions—such as rigid structural templates (Hyland, 2019) and predominantly corrective feedback practices (e.g., Shang, 2022; Zhang, 2017)—tend to treat uncertainty as a problem to be minimized in the pursuit of error-free performance. However, research has shown that strongly error-oriented approaches may inadvertently foster avoidance, leading learners to reduce linguistic risk-taking and rely on overly simplified structures in order to evade correction (Mahfoodh, 2017; Truscott, 1996). In this way, an excessive emphasis on certainty may constrain learner agency and suppress exploratory engagement with language (Larsen-Freeman, 2019; Lee, 2017; Ryan and Deci, 2020). These limitations highlight the need for pedagogical approaches that reframe linguistic uncertainty not as a source of threat, but as a productive and motivating condition for learning.

Generative artificial intelligence (GenAI), particularly text-to-image (T2I) models, offers a promising multimodal pathway for operationalizing such constructive uncertainty in EFL writing (Su and Yang, 2023; Wang et al., 2025). In AI-supported visual storytelling tasks, learners use English prompts to generate images that represent narrative ideas. Because the visual output is often partially unpredictable, the interaction introduces an externalized and potentially engaging form of uncertainty. Rather than intensifying anxiety about grammatical correctness, this uncertainty may encourage learners to participate in an iterative text-to-image cycle. When the generated image does not align with their intended meaning, learners are prompted to refine their input by searching for more precise vocabulary, clarifying semantic relations, and adjusting syntactic forms. From this perspective, T2I AI can function as a scaffold that redirects learners' attention away from fear of error and toward the intrinsically meaningful goal of producing a desired visual representation (Deci and Ryan, 2000). In doing so, it may reshape both the cognitive and affective dynamics of EFL writing.

Against this background, the present study adopts a quasi-experimental design to examine how a T2I AI-supported visual narrative intervention influences the affective, motivational, and behavioral outcomes of non-English-major EFL students. By comparing this multimodal approach with traditional text-only writing instruction, the study addresses the following research questions (RQs):

- RQ1: To what extent does the T2I AI-supported visual narrative intervention reduce EFL writing anxiety compared with traditional text-only writing?
- RQ2: How does this intervention affect students' writing motivation in EFL contexts compared with traditional text-only writing?
- RQ3: How does the intervention influence students' EFL writing performance compared with traditional text-only writing?
- RQ4: What cognitive and affective dynamics do students experience during the iterative “text input–image generation–prompt refinement” cycle, and how does AI-generated visual feedback mediate uncertainty within this process?

## 2 Literature review

### 2.1 The nature of uncertainty and anxiety in EFL writing

From an information-theoretic perspective, ambiguity in EFL writing can be understood as a form of psychological entropy, a state in which learners' cognitive systems are challenged by competing linguistic options and uncertain outcomes (Hirsh et al., 2012). In such high-entropy environments, learners must make continual decisions about vocabulary, syntax, organization, and meaning. When they are unable to manage or tolerate this ambiguity, intolerance of uncertainty (IU) becomes a key cognitive vulnerability that intensifies emotional distress (Carleton et al., 2012; Gu et al., 2020).

In second language learning, this distress often takes the form of FLWA. FLWA is a multidimensional affective construct that includes cognitive anxiety, somatic symptoms, and avoidance tendencies, all of which can interfere with learners' ability to process and produce language effectively (Xie and Yuan, 2020). Learners with high levels of writing anxiety are more likely to interpret the uncertainty of the writing process—especially the challenge of beginning with a blank page—not as an opportunity for exploration, but as a threat to their competence. As a result, their intrinsic motivation and writing self-efficacy may decline substantially (Tsao et al., 2017).

Traditional pedagogical responses to writing anxiety have often focused on reducing uncertainty through extensive corrective feedback (CF). This approach is based on the assumption that direct and comprehensive feedback reduces ambiguity and thereby improves linguistic accuracy. However, recent work adopting an ecological perspective on student emotions in feedback processes suggests that the effects of CF are far more complex (Chen and Nieminen, 2024). In particular, learners' emotional responses to feedback depend heavily on their pre-existing levels of anxiety (Rassaei, 2015, 2023). For highly anxious students, comprehensive correction may be experienced less as instructional support than as evaluative pressure. Under such conditions, learners may become overly dependent on direct feedback and excessively concerned with eliminating surface-level errors, often at the expense of deeper linguistic exploration and self-regulated learning (Xie and Yuan, 2020).

As a result, the traditional pursuit of certainty in EFL writing through rigid feedback practices may produce paradoxical effects. Rather than empowering learners, it can reinforce avoidance behaviors, leading them to minimize linguistic risk and rely on overly simple or “safe” structures to reduce the likelihood of further correction (Zhang and Rahimi, 2014). This tension points to a fundamental limitation in conventional writing pedagogy: when uncertainty is treated solely as a problem to be eliminated rather than a natural condition of language use to be navigated, pedagogical practices may inadvertently suppress learner agency and perpetuate a cycle of anxiety and avoidance.

### 2.2 Motivational dynamics and learner agency

Addressing the anxiety associated with linguistic uncertainty requires more than reducing error; it also requires restoring learner agency. Self-Determination Theory (SDT) offers a useful framework for understanding this process. According to SDT, motivation exists along a continuum from controlled to autonomous and is shaped by the extent to which three basic psychological needs are fulfilled: autonomy, competence, and relatedness (Chen and Jang, 2010; Teixeira et al., 2020). In learning environments where uncertainty is high but support is limited, learners often experience diminished autonomy and competence, which can lead to amotivation or reliance on externally regulated forms of engagement (Englund et al., 2023). By contrast, autonomy-supportive environments enable learners to exercise greater control over their learning and to interpret uncertainty not as a source of helplessness, but as a manageable and potentially productive challenge (Lu and Mohd Rameli, 2025).

From this perspective, reducing writing anxiety requires a pedagogical shift away from control-oriented, error-dominated instruction and toward forms of support that help learners reclaim ownership of the writing process. One promising direction lies in the design of scaffolded, iterative learning environments. Research on interactive and game-based learning has shown that carefully structured scaffolding can reduce cognitive overload while sustaining intrinsic motivation during complex tasks (Faber et al., 2024; Rienties et al., 2012). Importantly, these environments do not eliminate uncertainty; rather, they reshape its psychological function. In contrast to the anxiety-provoking uncertainty often associated with formal academic evaluation, interactive environments frequently rely on moment-to-moment uncertainty—for example, immediate but unpredictable feedback—to stimulate curiosity, experimentation, and persistence (Kumari et al., 2019).

This distinction has important implications for EFL writing. When ambiguous writing tasks are framed as opportunities for iterative exploration rather than as tests of correctness, learners may respond to uncertainty in more adaptive ways. In such settings, uncertainty no longer necessarily threatens perceived competence. Instead, when mediated through timely feedback and low-stakes experimentation, it can encourage learners to take linguistic risks, revise their ideas, and remain engaged in the writing process. Reframing uncertainty in this way may therefore be essential to fostering more autonomous and resilient writing behaviors.

### 2.3 Multimodal interventions and AI-generated visual content

One approach that may help transform uncertainty from a source of anxiety into a driver of exploration is digital multimodal composing (DMC), which has gained increasing attention in EFL education (Zhang et al., 2023). Drawing on Dual Coding Theory (Paivio, 1991) and cognitive load research (Sweller, 2011), multimodal learning is generally understood to be more effective when verbal and visual information are processed through complementary cognitive channels. In text-only writing, linguistic ambiguity can place heavy demands on working memory, particularly for learners with limited language proficiency. By contrast, multimodal environments provide visual support that can function as an external cognitive anchor, helping learners organize ideas, reduce processing burden, and translate abstract meanings into more concrete representations (Liang and Lim, 2021).

Recent developments in generative artificial intelligence (GenAI) have expanded the pedagogical possibilities of DMC by enabling the rapid production of AI-generated visual content (AIGVC). A recent systematic review suggests that the use of AIGVC has grown rapidly across educational settings, particularly in activities involving student engagement, creativity, and visual storytelling (Wang et al., 2024, 2025). In language learning contexts, text-to-image tools such as DALL·E and Midjourney allow learners to transform written prompts into visual outputs almost instantly, thereby creating new opportunities for multimodal meaning-making. Emerging empirical studies have begun to document the educational value of these tools. For example, Liu et al. (2024) found that GenAI-assisted DMC reshaped students' composing processes and generated patterns of text production that differed from those observed in conventional writing tasks. Jiang and Lai (2025) further reported that GenAI-supported composing enhanced students' sense of communicative purpose and improved the rhetorical quality of their multimodal work. Similarly, Jiang (2024) showed that GenAI can support learners in addressing complex design challenges by providing immediate visual inspiration.

Despite these advances, an important gap remains in the literature. Existing studies have focused primarily on the quality of final multimodal products (Jiang and Lai, 2025), broader cognitive changes in the composing process (Jiang, 2024; Smith et al., 2025), or the mechanics of cross-modal meaning transformation such as text-to-video resemiotization (Li et al., 2025). Much less attention has been paid to the affective and motivational dynamics embedded in the prompt-generation process itself. In particular, few studies have examined how the iterative text-to-image cycle may reshape learners' experience of uncertainty during EFL writing. Therefore, this study examines this cycle as a form of AI-generated visual feedback through an affective and motivational lens, aiming to explore whether and how it transforms linguistic uncertainty from a source of anxiety into a catalyst for autonomous engagement.

## 3 Method

### 3.1 Participants

This study adopted a quasi-experimental design with cluster-level random assignment and was conducted at a comprehensive university in China. The participants were 78 second-year undergraduate students enrolled in a compulsory EFL writing course. All participants were non-English majors and were recruited from two intact parallel classes taught by the same instructor, who had more than 5 years of experience in EFL teaching. The two classes were randomly assigned at the class level to either the experimental group (AI-assisted visual narrative group; *n* = 39) or the control group (traditional text-only writing group; *n* = 39). Because randomization was performed at the cluster (i.e., intact class) level rather than at the individual student level, the present design is more precisely characterized as a quasi-experimental cluster-randomized design rather than a fully randomized experiment (Shadish et al., 2002). The experimental group included 24 females and 15 males, aged 18 to 20 years (*M* = 19.00, SD = 0.79). The control group consisted of 22 females and 17 males, aged 18 to 21 years (*M* = 19.38, SD = 1.07). To evaluate the adequacy of the sample size, an *a priori* power analysis was conducted using G^*^Power 3.1 (Faul et al., 2009). For a one-way ANCOVA with two groups and one covariate, assuming a medium-to-large effect size (*f* = 0.30), an alpha level of 0.05, and a statistical power of 0.80, the minimum required sample size was estimated at 90 participants. Although the current sample of 78 falls slightly below this estimate, it exceeds the minimum requirement for detecting large effects (*f* = 0.40, required *N* = 52). Given that the primary hypotheses concerned anxiety and motivation—domains in which prior intervention studies have reported medium-to-large effects (e.g., Su and Yang, 2023; Zhang et al., 2023)—the present sample size was considered acceptable. Nonetheless, the results involving smaller effect sizes should be interpreted with caution, as discussed in the Limitations section. Prior to the intervention, informed consent was obtained from all participants. The consent form described the study's purpose, procedures, data collection methods, the use of the Doubao AI platform, and participants' right to withdraw at any time without academic penalty.

### 3.2 Experimental process

**Figure 1:**
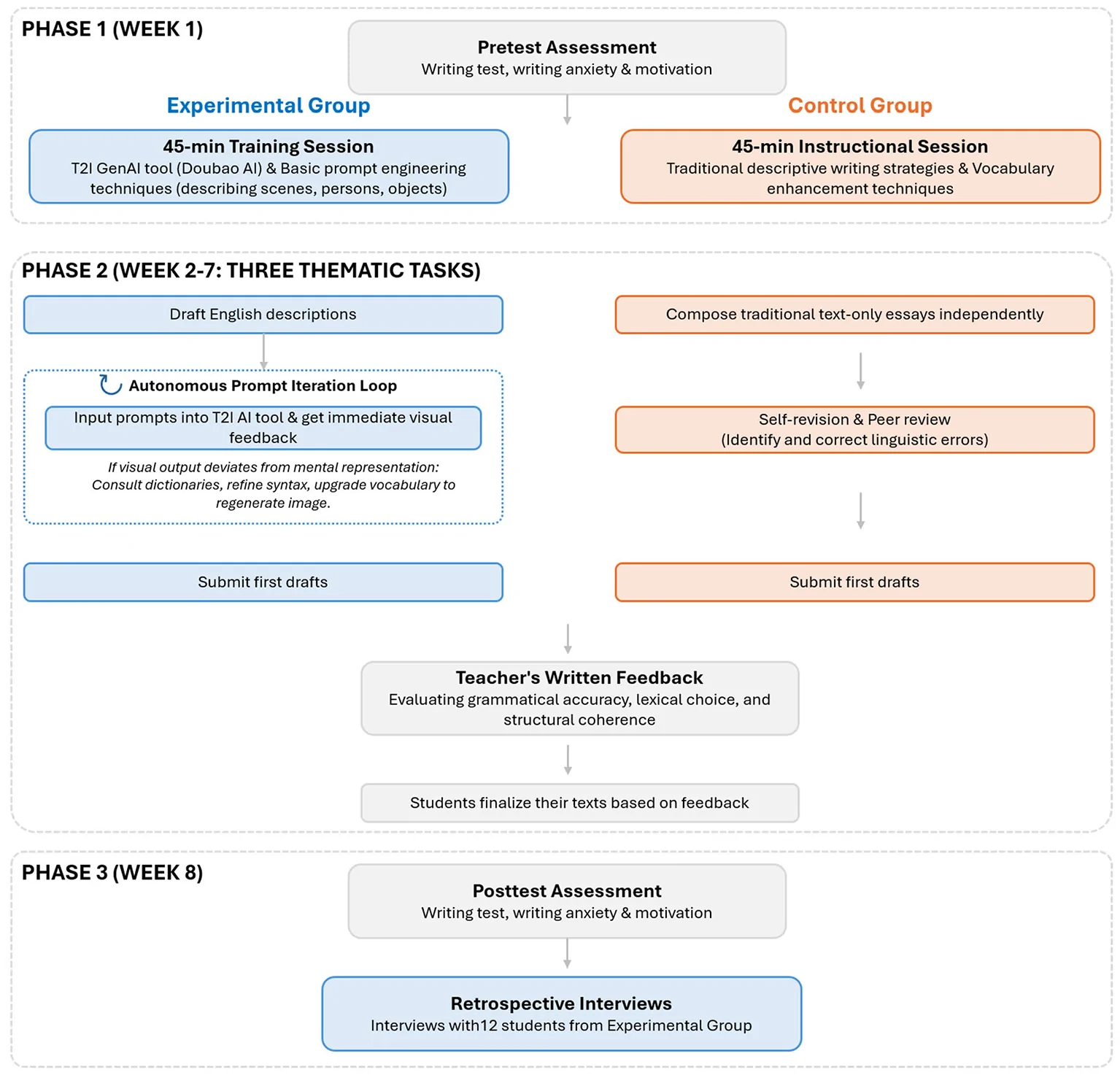
The experimental process.

The intervention lasted 8 weeks and consisted of three phases (see Figure 1). In Phase 1 (Week 1), all participants completed pre-intervention questionnaires measuring writing anxiety and writing motivation, as well as a writing test. Afterward, the experimental group received a 45-min training session on the use of a T2I GenAI tool (Doubao AI) and basic prompt-engineering strategies. The training focused on how to describe scenes, people, and objects in English and how to formulate effective prompts. Students were also explicitly instructed that no text-generation AI tools (e.g., ChatGPT, Wenxin Yiyan) were permitted at any point during the study; only the designated T2I tool (Doubao AI) was authorized for image generation. In parallel, the control group received a 45-min instructional session reviewing conventional descriptive writing strategies and vocabulary development techniques.

**Figure 2:**
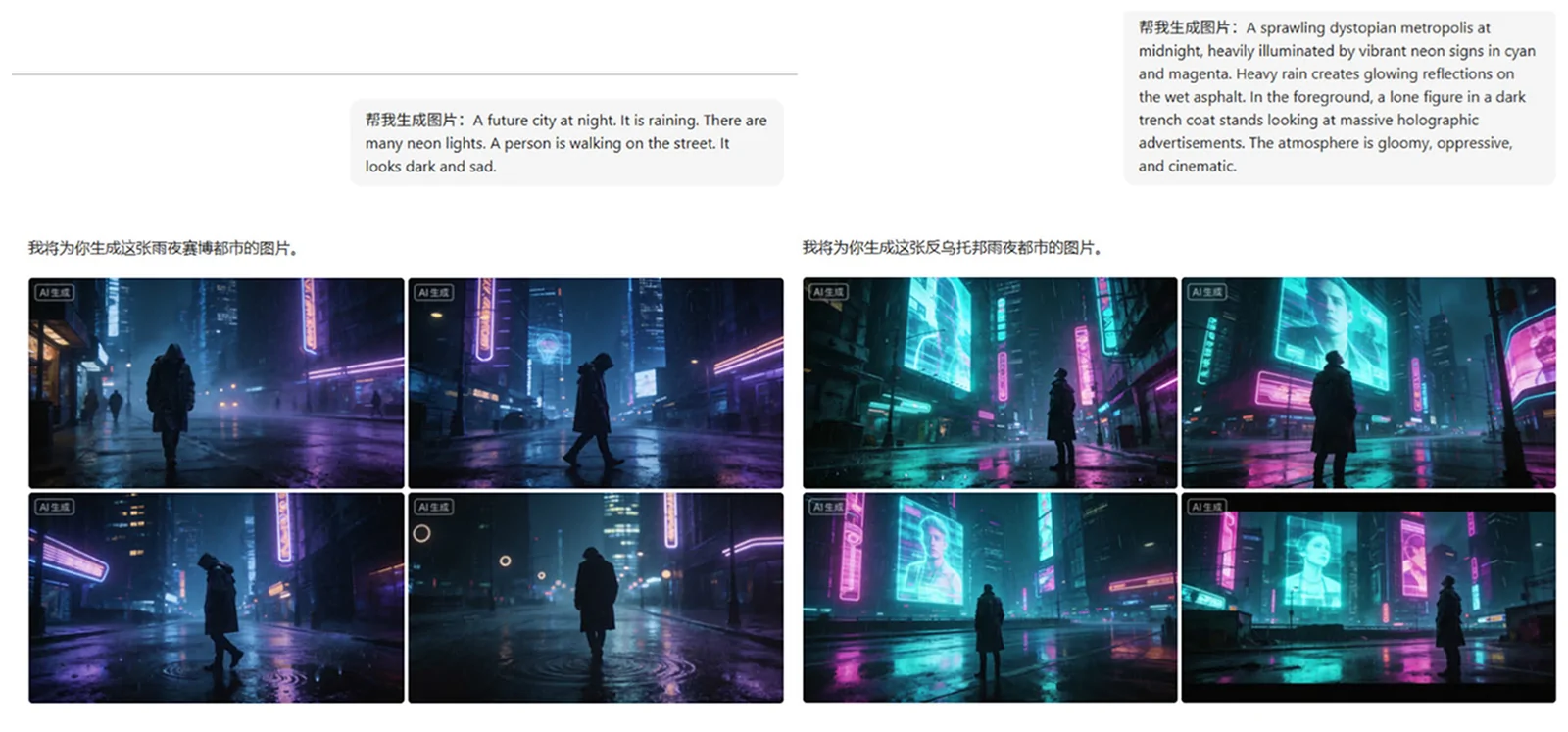
An example of a student's iterative prompt refinement process in the text-to-image (T2I) generation task.

Phase 2 (Weeks 2–7) involved three themed descriptive and narrative writing tasks, each of which lasted 2 weeks: my dream city, A Memorable Journey, and Life in 2050. Each week, both groups attended two 45-min writing sessions (totaling 90 min per week and 12 sessions across Phase 2), ensuring equivalent time-on-task between conditions. The drafting and revision procedures differed substantially across the two groups. In the experimental condition, participants were asked to create digital multimodal storyboards. The T2I tool used was Doubao AI (ByteDance, Seedream 4.0), with default image-generation parameters. Students accessed the tool via [their personal smartphones / classroom computers] under the supervision of the instructor. During the initial drafting stage, students wrote English descriptions and entered them as prompts into the T2I AI tool. Based on the visual feedback generated by the system, they then engaged in an autonomous process of prompt revision. When the generated image did not match their intended meaning or mental representation, students independently consulted dictionaries, revised sentence structures, and enriched their descriptive vocabulary before regenerating the image. This iterative process continued until they were satisfied with the visual output. Across the three tasks, students generated a variety of prompt iterations per task (range: 8–35). An example of this process is presented in Figure 2. After completing the multimodal drafting stage, students submitted their integrated text-and-image storyboards. In the control condition, participants completed conventional text-only essays on the same topics and were allocated the same total session time as the experimental group. During drafting, they composed their essays independently without multimodal support, using printed dictionaries and vocabulary reference sheets as permitted resources. After completing the first draft, they engaged in self-revision and peer review using a structured checklist covering grammatical accuracy, lexical variety, and content coherence to identify and correct linguistic problems before submission. This parallel structure ensured that any observed group differences could not be attributed to unequal time-on-task, resource availability, or instructor contact. To maintain ecological validity, both groups received written teacher feedback after submitting their drafts. The teacher commented on grammatical accuracy, lexical choice, and organizational coherence. Students in both groups then revised and finalized their texts in response to this feedback. By positioning AI-supported iteration before teacher feedback, the intervention was designed to function as a formative cognitive and affective support during the drafting process rather than as a substitute for explicit language instruction.

To ensure that students in the experimental group did not use text-generation AI tools to produce their written content, several verification measures were implemented. First, during in-class writing sessions, the instructor monitored students' screens to confirm that only Doubao AI's image-generation function was in use. Second, students were required to submit complete prompt-revision logs documenting each iteration of their text input and the corresponding AI-generated image. Third, all final written submissions were checked using a plagiarism detection system to screen for AI-generated text. No instances of unauthorized AI text generation were identified during the study.

In Phase 3 (Week 8), participants in both groups completed the post-intervention questionnaires on writing anxiety and motivation, as well as a post-test writing task. In addition, 12 students from the experimental group were selected through purposive stratified sampling for semi-structured retrospective interviews. To ensure maximum variation in perspectives, participants were stratified by their posttest writing anxiety levels: four students with high anxiety (top quartile), four with moderate anxiety (middle quartiles), and four with low anxiety (bottom quartile). Each interview was conducted individually in Chinese, lasted approximately 20–30 min, and was audio-recorded with the participant's consent. A semi-structured interview protocol was used, covering topics such as students' emotional experiences during the text-to-image iteration process, perceived changes in their approach to writing, and any challenges they encountered. The interviews aimed to explore their perceptions of using T2I GenAI tools during the intervention.

### 3.3 Measures

#### 3.3.1 EFL writing anxiety

EFL writing anxiety was measured using an adapted version of Cheng's (2004) Second Language Writing Anxiety Inventory (SLWAI). The SLWAI is a widely validated instrument designed to assess the anxiety learners experience specifically when writing in a second or foreign language. The instrument was administered in its original English form, as all participants had attained CET-4 and were enrolled in an English-medium writing course. No cross-linguistic translation was required. To suit the present research context, minor contextual wording modifications were made (e.g., replacing “English compositions” with “English writing tasks”) to align the items with the specific course activities. These modifications did not alter the construct coverage or the item structure of the original scale. The validity of the original SLWAI has been well established in East Asian EFL contexts (e.g., Cheng, 2004). The instrument contains 22 items distributed across three subscales: cognitive anxiety, somatic anxiety, and avoidance behavior. Participants responded on a 5-point Likert scale ranging from one (strongly disagree) to five (strongly agree). In the present study, the scale demonstrated excellent internal consistency (Cronbach's α = 0.91).

#### 3.3.2 Writing motivation

Students' writing motivation was assessed using a contextually adapted version of the Situational Motivation Scale (SIMS; Guay et al., 2000). Grounded in SDT, the SIMS is designed to measure individuals' state motivation during a specific activity at a particular moment. In this study, it was used to capture motivational changes during the writing and prompt-iteration tasks. As with the SLWAI, the SIMS was administered in English without cross-linguistic translation. Minor contextual modifications were made to the situational anchors so that each item referred specifically to the EFL writing activities used in this course (e.g., replacing the generic activity reference with “the writing task”). The scale consists of 16 items equally divided across four subscales representing the self-determination continuum: intrinsic motivation, identified regulation, external regulation, and amotivation. Responses were recorded on a 7-point Likert scale ranging from 1 (strongly disagree) to 7 (strongly agree). The instrument showed strong internal consistency in the present sample (Cronbach's α = 0.89). Because the SIMS measures state-level motivation during a specific activity rather than stable dispositional motivation, the term “writing motivation” is used throughout this manuscript as shorthand for “situational writing motivation” unless otherwise specified.

### 3.4 Ethical considerations for AI use

Given that the intervention involved students' interaction with a commercial generative AI platform (Doubao AI), several measures were taken to safeguard participant privacy and ensure responsible use. First, students were explicitly instructed to enter only English descriptive text prompts related to the assigned writing tasks. They were prohibited from inputting any personally identifiable information (e.g., names, student ID numbers, phone numbers, or photographs of themselves or others) into the platform. Second, during all in-class writing sessions, the instructor monitored students' interactions with the AI tool to ensure that no sensitive, offensive, or personally identifiable content was generated. No instances of inappropriate image generation were observed during the study. Third, all AI-generated images submitted as part of students' multimodal storyboards were stored on a university-secured drive and were accessible only to the research team. Upon completion of the study, students were informed that they could request the deletion of any AI-generated images associated with their work. Finally, at the beginning of the intervention (Phase 1), all participants received a brief instructional session on the responsible use of AI tools, emphasizing ethical guidelines such as avoiding the generation of harmful, violent, or discriminatory content and respecting intellectual property.

### 3.5 Data analysis

To address RQ1 and RQ2, which concerned the effects of the intervention on EFL writing anxiety and motivation, a two-step statistical procedure was employed. First, assumption checks were conducted to ensure that the data met all prerequisites for ANCOVA: (a) normality of residuals, assessed via the Shapiro–Wilk test; (b) homogeneity of error variances, assessed via Levene's test; (c) linearity between the covariate (pretest) and the dependent variable (posttest), assessed via Pearson correlations and scatterplot inspection; (d) homogeneity of regression slopes, assessed by testing the group × covariate interaction; and (e) absence of influential outliers, assessed via Cook's distance. Additionally, although independence of observations is a core ANCOVA assumption, the use of intact classes as the unit of assignment introduces potential non-independence at the cluster level; this constraint is discussed in the Limitations section. Next, independent-samples *t-*tests were performed on the pre-test anxiety and motivation scores to establish baseline equivalence between the experimental and control groups. Subsequently, separate one-way analyses of covariance (ANCOVAs) were conducted to examine post-test group differences. In these models, post-test anxiety and motivation scores served as the dependent variables, group assignment (AI-assisted vs. traditional) was entered as the fixed factor, and the corresponding pre-test scores were included as covariates. This procedure made it possible to estimate the net effect of the intervention while controlling for initial differences.

To address RQ3, students' writing performance was evaluated through both analytic scoring and inferential statistical analysis. All pre-test and post-test writing samples were anonymized, shuffled across time points, and coded with random identifiers so that the two raters were blinded to both group assignment and assessment occasion. The samples were then independently rated by two experienced EFL instructors. A researcher-developed 100-point analytic rubric was used, comprising four dimensions: lexical richness (30 points), syntactic complexity (30 points), descriptive detail and content development (30 points), and language mechanics (10 points). The scoring rubric can be seen in Table A1 in the Appendix. Inter-rater reliability was assessed using the intraclass correlation coefficient (ICC). The ICC was 0.80 for the pre-test and 0.85 for the post-test, indicating good agreement (Koo and Li, 2016). When the two raters' scores differed by more than 10 points on a given essay, they discussed the script to reach a consensus. If disagreement remained, a third senior EFL instructor served as adjudicator. After the final scores were determined, the same statistical procedure used for the affective variables was applied: an independent-samples *t*-test was first conducted on the pre-test scores to verify initial equivalence, followed by a one-way ANCOVA on the post-test scores with pre-test writing performance as the covariate.

To address RQ4, which focused on the micro-level mechanisms through which AI-generated visual feedback mediated uncertainty during prompt iteration, a qualitative thematic analysis was conducted on the retrospective interview data. The analysis followed Braun and Clarke's (2006) six-phase framework to ensure rigor and interpretive depth.

First, all interview recordings were transcribed verbatim in Chinese by the first author. The transcripts were then read repeatedly by both the first author and an independent coder (a doctoral researcher in applied linguistics with experience in qualitative analysis) to facilitate familiarization with the data. Next, meaningful segments related to students' experiences of uncertainty, emotional responses, challenges, and prompt-refinement strategies were independently assigned initial open codes by both coders. Inter-coder reliability was assessed on the full set of transcripts using Cohen's kappa (κ = 0.89), indicating great agreement (Landis and Koch, 1977). Discrepancies in coding were resolved through discussion between the two coders. These codes were subsequently grouped into broader candidate themes that captured the psychological and behavioral functions of AI-generated visual feedback. The themes were then reviewed and refined in relation to both the coded extracts and the full dataset to ensure that they accurately represented participants' accounts. Data saturation was considered achieved by the tenth interview, as no substantially new codes emerged from the final two transcripts (Guest et al., 2006). Finally, each theme was clearly defined and named, and representative quotations were selected and translated into English by the first author. To ensure translation accuracy, all translated quotations were independently back-checked by a bilingual colleague with expertise in EFL research. The original Chinese transcripts are available from the first author upon request. Following transcription and initial analysis, a member-checking procedure was carried out: each interviewee received a summary of their key themes and was invited to confirm, clarify, or modify the interpretation. All 12 participants confirmed the accuracy of the thematic summaries.

## 4 Results

### 4.1 Writing anxiety

An independent-samples *t*-test was first conducted to examine whether the experimental and control groups differed in EFL writing anxiety at pretest. As shown in Table 1, the experimental group reported a mean score of 3.800 (SD = 0.577), whereas the control group reported a mean score of 3.798 (SD = 0.573). The results indicated no statistically significant difference between the two groups, *t* = 0.014, *p* = 0.989. This finding suggests that both groups started the study with similarly high levels of writing anxiety.

**Table 1:** The results of the independent samples *t*-test that compares students' writing anxiety in the pretest between the experimental and control groups.

Group	*n*	Mean	SD	*t*	*p*
Experimental	39	3.800	0.577	0.014	0.989
Control	39	3.798	0.573		

Prior to conducting the ANCOVAs, all specific assumptions were verified for each dependent variable. The Shapiro–Wilk tests on the ANCOVA residuals confirmed normality for all three outcomes (anxiety: *W* = 0.988, *p* = 0.696; motivation: *W* = 0.995, *p* = 0.991; performance: *W* = 0.987, *p* = 0.631). Levene's tests indicated homogeneity of posttest variances across groups (anxiety: *F* = 0.051, *p* = 0.822; motivation: *F* = 0.692, *p* = 0.408; performance: *F* = 0.074, *p* = 0.787). Pearson correlations confirmed significant linear relationships between pretest and posttest scores in both groups for all three variables (all *r*s ≥ 0.284, all *p*s ≤ 0.080), with the exception of a marginally non-significant correlation for motivation in the control group (*r* = 0.284, *p* = 0.080); given that the overall correlation was significant (*r* = 0.347, *p* = 0.002) and ANCOVA is robust to minor deviations from strict linearity (Huitema, 2011), this was not considered problematic. The group × covariate interactions were non-significant for all three variables (anxiety: *F*(1, 74) = 0.512, *p* = 0.477; motivation: *F*(1, 74) = 1.296, *p* = 0.259; performance: *F*(1, 74) = 3.372, *p* = 0.070), confirming homogeneity of regression slopes. Finally, no influential outliers were identified; all Cook's distance values were well below the critical threshold of 1.0 (maximum *D* = 0.100, 0.107, and 0.099 for anxiety, motivation, and performance, respectively), and no cases had studentized residuals exceeding ±3 for anxiety or motivation (one case exceeded ±3 for performance but did not meet the Cook's *D* criterion for influence).

To assess the effect of the intervention, an ANCOVA was then conducted on posttest writing anxiety, with pretest scores entered as the covariate. As presented in Table 2, the adjusted posttest mean was 2.900 (SE = 0.085) for the experimental group and 3.750 (SE = 0.085) for the control group. The ANCOVA revealed a statistically significant group difference, *F* = 49.641, *p* < 0.001. These results indicate that students who participated in the T2I AI-supported visual narrative intervention experienced significantly lower levels of EFL writing anxiety than those who engaged in traditional text-only writing.

**Table 2:** The results of the ANCOVA that compares students' writing anxiety in the posttest between the experimental and control groups.

Group	*n*	Mean	SD	Adjusted mean	SE	*F*	*p*	partial η^2^
Experimental	39	2.900	0.600	2.900	0.085	49.641	<0.001	0.398
Control	39	3.750	0.588	3.750	0.085			

### 4.2 Writing motivation (situational)

An independent-samples *t*-test was conducted to compare the two groups' EFL situational writing motivation at pretest. As shown in Table 3, the experimental group had a mean score of 4.100 (SD = 0.679), while the control group had a mean score of 4.000 (SD = 0.780). The test results showed no statistically significant difference between the groups, *t* = 0.604, *p* = 0.548, indicating that they began the intervention with comparable levels of writing motivation.

**Table 3:** The results of the independent samples *t*-test that compares students' writing motivation in the pretest between the experimental and control groups.

Group	*n*	Mean	SD	*t*	*p*
Experimental	39	4.100	0.679	0.604	0.548
Control	39	4.000	0.780		

An ANCOVA was subsequently performed to compare posttest writing motivation, using pretest motivation as the covariate. As reported in Table 4, the adjusted mean for the experimental group was 5.182 (SE = 0.102), compared with 4.118 (SE = 0.102) for the control group. The analysis revealed a statistically significant difference, *F* = 54.322, *p* < 0.001. This finding suggests that the T2I AI-supported visual narrative intervention significantly enhanced students' EFL situational writing motivation relative to traditional text-only writing.

**Table 4:** The results of the ANCOVA that compares students' writing motivation in the posttest between the experimental and control groups.

Group	*n*	Mean	SD	Adjusted mean	SE	*F*	*p*	partial η^2^
Experimental	39	5.200	0.628	5.182	0.102	54.322	<0.001	0.420
Control	39	4.010	0.740	4.118	0.102			

### 4.3 EFL writing performance

An independent-samples *t*-test was used to determine whether the two groups differed in EFL writing performance at pretest. As shown in Table 5, the experimental group obtained a mean score of 69.449 (SD = 13.160), whereas the control group obtained a mean score of 72.497 (SD = 11.137). The difference was not statistically significant, *t* = 1.104, *p* = 0.273, indicating that the two groups had comparable writing proficiency prior to the intervention.

**Table 5:** The results of the independent samples *t*-test that compares students' writing performance in the pretest between the experimental and control groups.

Group	*n*	Mean	SD	*t*	*p*
Experimental	39	69.449	13.16	1.104	0.273
Control	39	72.497	11.137		

To examine post-intervention differences, an ANCOVA was conducted with posttest writing performance as the dependent variable and pretest performance as the covariate. As shown in Table 6, the adjusted posttest mean was 83.854 (SE = 1.523) for the experimental group and 79.184 (SE = 1.523) for the control group. The ANCOVA yielded a statistically significant group effect, *F* = 4.663, *p* = 0.034, although the effect size was small (partial η^2^ = 0.059). These results indicate that students in the T2I AI-supported visual narrative condition showed a statistically significant but modest advantage over those in the traditional text-only condition on EFL writing performance. It is worth noting that this effect was considerably smaller than those observed for writing anxiety (partial η^2^ = 0.398) and motivation (partial η^2^ = 0.420), suggesting that the intervention had a stronger impact on affective outcomes than on linguistic output within the 8-week timeframe. In terms of practical significance, the adjusted mean difference of 4.67 points on a 100-point analytic rubric (Cohen's *d* = 0.49) corresponds to approximately one performance level on a single rubric dimension (e.g., moving from “Adequate” to “Good” in lexical richness; see the Appendix). It is also worth noting that both groups improved substantially from pretest to posttest (experimental group: from 69.45 to 82.96, a gain of 13.51 points; control group: from 72.50 to 80.08, a gain of 7.58 points), indicating that the traditional writing instruction also produced meaningful progress. The between-group difference therefore reflects a differential rate of improvement rather than a contrast between progress and stagnation. While this differential gain is statistically significant, its practical importance should be interpreted in light of the modest effect size, and readers should note that statistical significance does not automatically imply educational meaningfulness (Plonsky and Oswald, 2014).

**Table 6:** The results of the ANCOVA that compares students' writing performance in the posttest between the experimental and control groups.

Group	*n*	Mean	SD	Adjusted mean	SE	*F*	*p*	partial η^2^
Experimental	39	82.959	11.480	83.854	1.523	4.663	0.034	0.059
Control	39	80.079	12.159	79.184	1.523			

### 4.4 Thematic analysis of students' experience

To further explore students' cognitive and affective experiences during the iterative cycle of text input, image generation, and prompt refinement, we conducted a thematic analysis of the retrospective interview data. As presented in Table 7, three overarching themes emerged: (1) transforming uncertainty from threat into playful exploration, (2) visual feedback as an autonomy-supportive scaffold, and (3) iterative agency and lexical risk-taking.

**Table 7:** The results of the thematic analysis.

Theme	Sub-codes	Description	Representative quotes
Transforming uncertainty from threat to playful exploration	Mitigation of blank-page anxiety	AI intervention removes the initial paralysis caused by linguistic uncertainty when facing a blank screen.	“I used to freeze when starting an essay. Now, knowing I just need a basic prompt to get a picture first, that initial panic is totally gone.” (Student A)
	Gamification of trial-and-error	The unpredictable nature of AI generation turns the writing process into a low-risk, blind-box game.	“It feels like a game to fix a ‘bug'. If the picture is wrong, I don't feel defeated; I just want to try another word to ‘unlock' the right image.” (Student B)
	Shift from evaluation fear to curiosity	Learners' attention shifts from worrying about teacher grading to anticipating the AI's visual interpretation.	“I wasn't thinking about grammar mistakes anymore. I was just super curious to see how the AI would draw the ‘dystopian' world I described.” (Student *F*)
Visual feedback as an autonomy-supportive scaffold	Instant gratification	The immediate visual reward satisfies the learner's need for competence and sustains engagement.	“Writing is usually so boring, but seeing my text instantly turn into a cool cyberpunk scene gave me a huge sense of achievement right away.” (Student E)
	Discrepancy-driven attention	The gap between the learner's mental imagery and the AI's output prompts focused linguistic reflection.	“I wrote 'a big building', but the AI drew a normal house. I realized my English was too vague, which made me really look at my words again.” (Student C)
	Non-judgmental mediation	AI provides objective visual consequences without the emotional weight of traditional human correction.	“The AI doesn't judge my poor vocabulary or put red crosses on my paper; it just shows me exactly what my words mean visually. It feels safe.” (Student D)
Iterative agency and lexical risk-taking	Autonomous vocabulary seeking	Driven by visual goals, students actively and voluntarily search for advanced, precise vocabulary.	“To make the city look scary, ‘bad' wasn't enough. I actively used a dictionary to find words like ‘oppressive' and ‘gloomy' because I wanted the exact vibe.” (Student H)
	Syntactic refinement for precision	Students independently modify sentence structures and prepositions to clarify relationships for the AI.	“The AI put the flying car behind the building instead of above it. I had to rewrite the prepositional phrases and use a relative clause to make it understand.” (Student G)
	Ownership of the creative process	The iterative cycle fosters a strong sense of agency and personal connection to the final multimodal text.	“By the time I got the perfect image after five tries, I felt like I truly owned both the picture and the English sentences I wrote. I was in control.” (Student I)
Perceived challenges and residual concerns	Frustration with AI misinterpretation	Persistent mismatch between intended meaning and AI-generated output across multiple iterations causes temporary discouragement.	“There were several times when the generated image was clearly inconsistent with what I had in mind.” (Student J)
	Time pressure and cognitive demand	The iterative prompt-refinement cycle is perceived as more time-consuming than traditional drafting, especially under deadline constraints.	“Going back and forth with the generation was quite time-consuming, especially when there was a deadline.” (Student K)
	Concern about transferability	Students question whether linguistic gains will persist in conventional writing tasks without visual AI support.	“I wonder whether my language improvement would still hold up if this visual AI were removed.” (Student L)

Under the first theme, transforming uncertainty from threat into playful exploration, three subthemes were identified: mitigation of blank-page anxiety, gamification of trial and error, and a shift from fear of evaluation to curiosity. The first subtheme captures how the intervention reduced the sense of paralysis often associated with beginning a writing task. One student explained that knowing a simple prompt could immediately generate an image largely eliminated the panic usually triggered by a blank page. The second subtheme reflects the way the unpredictability of AI-generated images made the writing process feel more like a low-stakes game than a formal assessment. As one participant noted, when the generated image was inaccurate, the experience felt less like failure and more like fixing a “bug” by trying a different word. The third subtheme describes a clear shift in students' attention: rather than worrying about grades or grammatical mistakes, they became curious about how the AI would visually interpret their ideas. One student, for example, reported focusing less on grammar and more on anticipating how the AI would depict the dystopian world they had described.

The second theme, visual feedback as an autonomy-supportive scaffold, included the subthemes instant gratification, discrepancy-driven attention, and non-judgmental mediation. Instant gratification refers to the immediate visual response produced by the AI, which appeared to strengthen learners' sense of competence and sustain engagement. One student described the experience of seeing their text instantly transformed into a cyberpunk scene as highly rewarding. Discrepancy-driven attention captures how mismatches between students' intended meaning and the AI's visual output directed their attention to linguistic precision. For instance, one participant realized that their description had been too vague when the AI generated an ordinary house rather than the more complex structure they had imagined, prompting them to reconsider their vocabulary choices. Non-judgmental mediation refers to the emotionally safer role played by the AI. Unlike teacher correction, which can be experienced as evaluative, the AI simply rendered students' language into images without overt judgment. One student emphasized that this made the process feel safe, because the AI did not criticize weak vocabulary or mark errors in red.

The third theme, iterative agency and lexical risk-taking, comprised autonomous vocabulary seeking, syntactic refinement for precision, and ownership of the creative process. Autonomous vocabulary seeking describes how students, motivated by the desire to generate more accurate or vivid images, independently searched for more precise and expressive language. One participant explained that they deliberately consulted a dictionary for words such as “oppressive” and “gloomy” rather than relying on generic descriptors like “bad.” Syntactic refinement for precision refers to students' efforts to revise sentence structure, prepositions, and clause relationships in order to communicate spatial or logical details more effectively to the AI. For example, one student reported rewriting prepositional phrases and adding a relative clause to ensure that a flying car appeared above a building rather than beside it. Finally, ownership of the creative process reflects students' growing sense of agency over both the visual and verbal dimensions of their work. As one participant noted, after several rounds of revision to achieve the desired image, they felt a strong sense of ownership not only of the final picture but also of the English sentences used to create it.

While the predominant interview responses reflected positive experiences, several students also reported challenges associated with the T2I iteration process. These less favorable perspectives formed a fourth theme: perceived challenges and residual concerns.

Three subthemes were identified. The first, frustration with AI misinterpretation, captures instances in which the AI-generated output persistently deviated from the learner's intended meaning despite multiple attempts at prompt revision. As one student reported, “There were several times when the generated image was clearly inconsistent with what I had in mind” (Student J). Such moments of repeated mismatch, while sometimes motivating further revision (as described under Theme 1), could also lead to temporary discouragement when accumulated over multiple iterations. The second subtheme, time pressure and cognitive demand, reflects students‘ perception that the iterative prompt-refinement cycle was more time-consuming than conventional drafting, particularly under deadline constraints. One participant noted, “Going back and forth with the generation was quite time-consuming, especially when there was a deadline” (Student *K*). This suggests that while the iterative process fostered linguistic engagement, it also imposed additional time costs that may not always be compatible with routine classroom schedules. The third subtheme, concern about transferability, captures students' uncertainty about whether the linguistic gains developed through the T2I process would persist in writing contexts without visual AI support. One student reflected, “I wonder whether my language improvement would still hold up if this visual AI were removed” (Student *L*). This concern highlights the importance of investigating the long-term retention and transfer of the skills cultivated through AI-supported writing tasks.

These critical perspectives indicate that the benefits of the T2I intervention were not without caveats. The challenges identified by students offer important considerations for instructional design, which are further discussed in the Implications section.

## 5 Discussion and conclusion

The current study investigated how a T2I AI-supported visual narrative intervention influences EFL learners' writing anxiety, motivation, and performance, alongside their micro-level cognitive and affective dynamics during the prompt iteration cycle. Based on an 8-week quasi-experimental design involving 78 undergraduate EFL learners, the findings offer several theoretical and pedagogical insights into the integration of GenAI in language learning.

### 5.1 Findings

The first major finding reveals that participants in the T2I visual narrative group exhibited lower levels of EFL writing anxiety at the posttest compared to their peers in the traditional text-only condition. This aligns with recent empirical research suggesting that DMC and generative AI can effectively lower learners' affective filters (Jiang and Hafner, 2024; Su and Yang, 2023; Zhang et al., 2023). Moving beyond attributing this reduction merely to technological novelty, this study highlights a deeper mechanism: AI visual storytelling may mitigate the psychological roots of writing anxiety by transforming threat-based uncertainty into exploratory uncertainty, at least within the intervention period.

This alleviating effect can be theoretically substantiated through the psychological uncertainty model (Gu et al., 2020) and dual coding theory (Paivio, 1991). In traditional text-only environments, confronting a blank screen often induces structural and lexical ambiguity, which, lacking immediate formative feedback, can trigger a fear of negative evaluation and subsequent avoidance behaviors (Cheng, 2004; Truscott, 1996). Conversely, the T2I model externalizes this linguistic ambiguity by converting it into immediate, non-judgmental visual feedback. Acting as an external cognitive anchor, the visual modality may have helped reduce the working memory load, thereby facilitating smoother meaning-making (Liang and Lim, 2021). However, it should be noted that this effect could also be partially attributable to the immediacy and non-evaluative nature of the AI feedback rather than to the visual modality alone. As qualitative data revealed, the unpredictability of AI outputs reframed the trial-and-error process. Rather than fixating on formal evaluation, learners redirected their attention toward seeing how the AI interpreted their ideas. Inaccurate image generations were perceived not as linguistic failures, but as iterative problem-solving opportunities, effectively dismantling the evaluative threat typically associated with L2 writing. However, two interpretive caveats are warranted. First, writing anxiety was assessed through self-report questionnaires, which capture perceived anxiety rather than objective physiological or behavioral indicators. Future studies incorporating psychophysiological measures (e.g., heart rate variability or skin conductance) would provide more robust evidence for anxiety reduction. Second, it is possible that part of the observed decrease in anxiety reflects a novelty effect associated with the introduction of a new technology rather than a sustained change in learners' affective orientation toward writing. Although the intervention spanned 8 weeks—longer than typical novelty windows reported in educational technology research—the absence of a delayed posttest means that the durability of this effect remains an open question.

In addition to reducing anxiety, the intervention fostered greater situational writing motivation. Students in the T2I group demonstrated stronger engagement than the control group, corroborating literature on the motivational affordances of gamified and multimodal learning environments (Jiang and Lai, 2025; Ratinho and Martins, 2023). Crucially, this study suggests that the iterative, agentic, and discrepancy-driven nature of the T2I generation process may have contributed to sustaining students' situational intrinsic motivation, though the relative contributions of visual feedback, AI immediacy, and non-judgmental interaction cannot be fully separated in the current design. Viewed through the lens of SDT, the environment effectively satisfied students' basic psychological needs for competence and autonomy (Chen and Jang, 2010; Teixeira et al., 2020). While delayed feedback in traditional drafting often thwarts a sense of competence, the T2I AI functioned as a responsive, autonomy-supportive scaffold. Instantaneous visual validation of their initial prompts reinforced learners' communicative efforts. Furthermore, the iterative loop—where students independently manipulated vocabulary and syntax to alter visual outputs—granted them considerable autonomy. The discrepancy between intended meaning and actual output transformed language refinement into a self-regulated puzzle, shifting learners' situational orientation from fulfilling external academic requirements to pursuing personally meaningful design goals (Lu and Mohd Rameli, 2025). Nevertheless, it should be acknowledged that the SIMS captures situational motivation at a particular moment in time, and the elevated motivation observed in the experimental group may be partially attributable to the novelty and gamified nature of the AI tool rather than to a fundamental shift in motivational orientation. The absence of a longitudinal follow-up or delayed posttest prevents definitive conclusions about whether these motivational gains would persist beyond the intervention period. Future research should include delayed assessments and gradual fading of the AI scaffold to disentangle sustained motivational change from transient novelty-driven engagement.

Finally, the T2I intervention was associated with a statistically significant, though modest, improvement in EFL writing performance (partial η^2^ = 0.059). Although both groups began with comparable proficiency, the experimental group scored significantly higher than the control group at the posttest, consistent with findings by Liu et al. (2024) and Xu (2026). However, the small effect size suggests that the writing performance gains, while statistically significant, were more limited in magnitude than the effects on anxiety and motivation. Beyond cognitive load reduction, this study explores how cross-modal translation may contribute to improving linguistic output quality, though the modest effect size (partial η^2^ = 0.059; Cohen's *d* = 0.49) warrants a cautious interpretation of this mechanism. This improvement is best explained by the output hypothesis (Swain, 2005) and the concept of “noticing the gap” (Schmidt, 1990). In text-only drafting, EFL learners frequently rely on superficial semantic processing without being challenged on linguistic precision. In the T2I environment, however, the AI serves as a literal visual interlocutor. When an AI-generated image deviates from a learner's mental representation, the salient visual mismatch compels the learner to notice gaps in their interlanguage repertoire.

Retrospective interviews confirmed that this realization triggered active lexical risk-taking. Rather than settling for basic descriptors, students deliberately consulted external resources for precise vocabulary (e.g., substituting bad with oppressive). According to Swain (2005), such communicative breakdowns force a shift from semantic to rigorous syntactic processing. To communicate spatial and logical nuances effectively, students proactively engaged in syntactic refinement, such as revising prepositional phrases and adding relative clauses for accurate visual placement. Consequently, this AI-mediated uncertainty organically stretched students' linguistic boundaries, contributing to the observed improvement in writing performance and **fostering** a stronger sense of ownership over their dual-modality creations. That said, the relatively modest effect on writing performance (compared with the large effects on anxiety and motivation) may reflect the fact that gains in linguistic accuracy and complexity require sustained practice to fully materialize (Norris and Ortega, 2000). The 8-week intervention may have been sufficient to shift affective dispositions but too brief for these shifts to translate into proportionally large gains in written output quality. Furthermore, the practical magnitude of the performance gain should be interpreted with care. A between-group difference of approximately 4.7 points, while statistically significant, falls within a range that could be influenced by factors such as rater variability or task difficulty. The effect size (Cohen's *d* = 0.49) is at the boundary between small and medium by conventional standards (Cohen, 1988) and is comparable to the median effect size reported in L2 instructional intervention meta-analyses (Plonsky and Oswald, 2014). Thus, while the T2I intervention appears to offer a modest advantage for writing development, the current evidence does not support strong claims about transformative effects on writing quality. Moreover, the current study lacks fine-grained process data—such as time-stamped prompt logs, number of iterations per sentence, or duration of each revision cycle—that would allow direct linking of specific iteration behaviors to measurable gains in writing quality. Without such data, the causal pathway from iterative prompt refinement to improved writing performance remains inferential rather than empirically traced. Future studies should incorporate screen-recording data, keystroke logging, or automated prompt-log analysis to provide more granular evidence of how the text-to-image iteration process contributes to linguistic development.

An important methodological caveat concerns the interpretation of the observed group differences. The current two-group design compared the T2I AI-supported intervention with a traditional text-only writing condition. However, the two conditions differed not only in the presence of visual feedback but also in the immediacy of feedback (instant AI-generated images vs. delayed teacher and peer responses during drafting), its affective tone (non-judgmental AI rendering vs. evaluative peer review), and its interactive nature (iterative human–AI prompt exchange vs. independent drafting). Consequently, the observed reductions in anxiety and gains in motivation and performance cannot be attributed exclusively to the visual or multimodal dimension of the intervention; they may be partially driven by the immediacy, interactivity, or non-evaluative character of the AI feedback. The qualitative data offer some indirect evidence for the specific role of visual feedback—for example, students' discrepancy-driven attention was triggered by visual mismatches between intended meaning and generated images, and the gamified experience was tied to the unpredictability of visual outputs rather than to textual responses. Nevertheless, in the absence of an active control group receiving immediate, non-judgmental text-based AI feedback (e.g., from a chatbot without visual output), the unique contribution of the visual modality cannot be causally isolated. Future research should therefore adopt a three-arm design—comparing T2I AI, text-only AI (e.g., chatbot feedback), and traditional instruction—to disentangle the specific effects of visual feedback from those of AI-mediated immediacy and interactivity more broadly.

### 5.2 Implications, limitations, and future research

#### 5.2.1 Theoretical implications

This study contributes to the theoretical understanding of uncertainty in L2 writing. Traditionally, L2 acquisition literature has predominantly viewed linguistic ambiguity as a psychological threat or a deficit that induces anxiety and impedes cognitive processing. However, by situating SDT within a technology-mediated environment, the present study reframes this perspective through the lens of uncertainty transformation. The findings demonstrate that when a T2I GenAI acts as a multimodal mediator, it can convert anxiety-inducing linguistic ambiguity into an exploratory uncertainty that actively drives intrinsic motivation. This transition broadens the conceptualization of AI visual feedback, moving beyond viewing it merely as a novel tool to understanding it as an autonomy-supportive scaffold that mitigates affective filters. Ultimately, these insights offer a grounded theoretical basis for how learner agency and motivation can be sustained in emerging human-AI co-creation contexts.

#### 5.2.2 Pedagogical implications

From a pedagogical perspective, the findings advocate for rethinking how EFL writing instruction is scaffolded. Rather than relying solely on the traditional, linear model—where text-only drafting is often followed by delayed teacher evaluation—educators can purposefully integrate T2I AI into the initial brainstorming and drafting stages. Designing prompt iteration tasks creates a low-stakes, non-judgmental space for linguistic trial-and-error, which can effectively alleviate early-stage writing anxiety.

Furthermore, this approach necessitates a gradual shift in the teacher's role from a primary evaluator of linguistic accuracy to a facilitator of multimodal meaning-making. At the same time, the challenges reported by some students—particularly frustration with persistent AI misinterpretation and the added time demands of the iterative process—underscore the need for adequate scaffolding and realistic time allocation. Instructors should anticipate that some learners may experience temporary frustration during early iterations, and should provide targeted guidance on prompt-engineering strategies to mitigate this. Additionally, the concern about transferability raised by several participants suggests that teachers should explicitly design bridging activities that help students apply the vocabulary and syntactic strategies developed through T2I tasks to conventional, non-AI-supported writing contexts. Teachers can encourage students to utilize the unpredictable nature of AI-generated images to consciously notice the gap in their interlanguage. By guiding learners to resolve the discrepancies between their intended meaning and the AI's visual output, educators can naturally stimulate lexical risk-taking and syntactic refinement. Pedagogical designs that position generative AI as a collaborative exploratory tool, rather than a mere shortcut to a final product, are more likely to maximize its affordances in language education.

#### 5.2.3 Limitations and future research

Several limitations of the current study warrant consideration and provide avenues for future research.

First, the findings are based on a specific cohort of 78 non-English major undergraduates at a single university in China, which limits the broader generalizability of the results. Because only two intact classes served as the units of assignment, the design does not permit a full estimation of between-cluster variance, and potential class-level confounds—such as pre-existing group dynamics or peer interaction effects—cannot be entirely ruled out, despite the established baseline equivalence on the measured variables. Moreover, the *a priori* power analysis indicated that the sample of 78 is adequate for detecting large effects but may be underpowered for small effects, as reflected in the relatively modest effect size for writing performance (partial η^2^ = 0.059). Future studies should consider multi-class designs involving a larger number of clusters and participants to allow for multilevel modeling, more robust causal inferences, and the detection of smaller effects. Extending this T2I visual narrative intervention to diverse EFL populations and institutional contexts would further strengthen the external validity of the findings.

Second, the sample size was insufficient for conducting confirmatory factor analysis to re-examine the factor structures of the measurement instruments within this specific sample (Kline, 2016). Although the contextual modifications to the SLWAI and SIMS were minor and did not alter the construct domains, and subscale-level reliability was satisfactory, future research with larger samples should verify the factor structures of these instruments when applied to similar EFL populations.

Third, while the 8-week quasi-experimental design captured meaningful shifts in anxiety, motivation, and writing performance, it does not fully account for the potential novelty effect of generative AI. The absence of a delayed posttest limits the ability to determine whether the observed gains are durable or primarily reflect transient novelty-driven engagement. Future longitudinal research should include delayed posttests (e.g., 4 to 8 weeks after the intervention) and, where feasible, a gradual withdrawal design to track whether autonomous writing behaviors and reduced anxiety levels persist once the AI visual scaffold is faded over an entire academic semester or year.

Fourth, the two-group design does not permit the isolation of the unique effect of the visual modality. The experimental condition differed from the control condition in multiple respects simultaneously: the presence of AI-generated visual feedback, the immediacy of that feedback, its non-evaluative tone, and the interactive prompt-refinement loop. Without an active control group receiving immediate, non-judgmental text-based AI feedback (e.g., a chatbot providing linguistic suggestions without visual output), the observed effects may reflect the combined influence of these features rather than the visual dimension specifically. Future studies should employ a three-arm design to disentangle the contributions of visual feedback, AI-mediated immediacy, and multimodal interaction.

Fifth, all affective and motivational variables in this study were assessed through self-report instruments, which are subject to social desirability bias and may not fully capture the complexity of learners' internal states. Similarly, the exploration of micro-level cognitive and affective dynamics relied on retrospective interview data. While independent dual coding and inter-coder reliability checks were employed to strengthen the rigor of the qualitative analysis, member checking was not systematically conducted. Future studies should triangulate self-report data with behavioral indicators (e.g., voluntary writing output, autonomous revision frequency) or physiological measures (e.g., heart rate variability), and should incorporate formal member-checking procedures to further enhance the credibility of the qualitative findings.

Finally, although the average number of prompt iterations per task was reported, the current study lacks fine-grained process-tracing data—such as time-stamped prompt-revision logs, screen recordings, and keystroke data—that would enable direct linking of specific iteration behaviors to measurable gains in writing quality. Future investigations should incorporate such multimodal process data to provide more granular evidence of how the text-to-image iteration process contributes to linguistic development.

## Data Availability

The raw data supporting the conclusions of this article will be made available by the authors, without undue reservation.
