# Supplementary material for: Transforming uncertainty into exploration: effects of text-to-image AI on EFL learners' writing anxiety, motivation, and performance
Source: Front Psychol. 2026 Aug 24;17:1866688. doi: 10.3389/fpsyg.2026.1866688 (PMC13547027; doi:10.3389/fpsyg.2026.1866688)
Supplement: Supplementary file 1 [file Supplementary_file_1.docx]

# Appendix

Table A1. Analytic Writing Rubric (100 Points)

| Dimension | Score | Level | Descriptors |
| --- | --- | --- | --- |
| Lexical Richness  (30 points) | 25–30 | Excellent | Demonstrates a sophisticated and varied vocabulary. Word choices are precise, contextually appropriate, and convey nuanced meaning (e.g., uses "oppressive," "dilapidated" rather than generic terms like "bad" or "old"). Employs a wide range of descriptive adjectives, adverbs, and domain-specific terms. Collocations and idiomatic expressions are used naturally. Minimal to no reliance on high-frequency basic vocabulary. |
|  | 19–24 | Good | Displays a broad vocabulary with generally precise word choices. Uses some varied and descriptive language that goes beyond basic vocabulary, though occasional imprecision or repetition may occur. Attempts to use lower-frequency or more expressive words, with mostly successful results. |
|  | 13–18 | Adequate | Shows an adequate vocabulary range sufficient to convey general meaning. Relies primarily on common, high-frequency words with limited variety. Some attempts at more expressive or precise vocabulary are evident but inconsistent. Occasional word choice errors that do not severely impede comprehension. |
|  | 7–12 | Limited | Displays a restricted vocabulary range. Relies heavily on basic, repetitive, and high-frequency words (e.g., "good," "big," "very"). Word choice errors are frequent and sometimes obscure intended meaning. Little evidence of lexical risk-taking or variety. |
|  | 1–6 | Very Limited | Extremely narrow and impoverished vocabulary. Word choices are frequently inappropriate or incorrect, significantly obscuring meaning. Heavy reliance on L1 transfer or invented words. Communication is severely hampered by lexical limitations. |
| Syntactic Complexity  (30 points) | 25–30 | Excellent | Exhibits a wide variety of sentence structures, including complex and compound-complex sentences. Effectively employs subordinate clauses (e.g., adverbial, relative, noun clauses), participial phrases, and varied sentence openings. Sentences are well-formed, fluent, and demonstrate sophisticated control of English syntax. Syntactic choices enhance meaning and readability. |
|  | 19–24 | Good | Uses a range of sentence types, including some complex structures with subordination and coordination. Sentence patterns show reasonable variety, though occasional structural awkwardness may occur. Most sentences are grammatically accurate and demonstrate competent syntactic control. |
|  | 13–18 | Adequate | Demonstrates a basic ability to construct both simple and compound sentences. Attempts at complex sentences are present but may contain structural errors (e.g., run-on sentences, comma splices, or fragmented clauses). Sentence patterns tend to be repetitive (e.g., predominantly S-V-O). |
|  | 7–12 | Limited | Relies predominantly on simple, short sentences with minimal variation. Attempts at compound or complex structures frequently result in grammatical errors. Sentence boundaries are often unclear. Syntactic range is noticeably constrained. |
|  | 1–6 | Very Limited | Produces only fragmented or incomplete sentences. Sentence structures are severely malformed, making the text difficult to follow. Little to no evidence of syntactic variety or control. |
| Descriptive Detail and Content Development  (30 points) | 25–30 | Excellent | Content is richly developed with vivid, specific, and well-elaborated descriptive details. Sensory language (visual, auditory, tactile, etc.) is used effectively to create a compelling scene or narrative. Ideas are logically organized with clear thematic coherence. The writing demonstrates a strong sense of purpose and audience awareness. Transitions between ideas are smooth and purposeful. |
|  | 19–24 | Good | Content is well developed with adequate descriptive details that support the main theme. Some use of sensory or figurative language is evident. Organization is generally clear and coherent, with appropriate transitions. Minor gaps in elaboration or occasional vagueness do not significantly detract from overall quality. |
|  | 13–18 | Adequate | Content addresses the topic but with limited elaboration. Descriptions tend to be general rather than specific (e.g., "The city was beautiful" without supporting details). Organization is discernible but may lack smooth transitions. Some ideas are underdeveloped or loosely connected. |
|  | 7–12 | Limited | Content is minimally developed with few descriptive details. The writing is vague, superficial, or list-like, lacking meaningful elaboration. Organizational structure is weak, with abrupt shifts between ideas. The reader must infer much of the intended meaning. |
|  | 1–6 | Very Limited | Content is extremely sparse, off-topic, or incoherent. Little to no descriptive detail is provided. There is no discernible organizational structure. The text fails to convey a clear scene, narrative, or central idea. |
| Language Mechanics  (10 points) | 9–10 | Excellent | Near error-free control of spelling, punctuation, and capitalization. Formatting conventions (e.g., paragraph indentation, consistent spacing) are observed throughout. Any errors are rare and do not affect readability. |
|  | 7–8 | Good | Generally accurate spelling, punctuation, and capitalization with only occasional minor errors. These errors do not impede comprehension or distract the reader. |
|  | 5–6 | Adequate | Some noticeable errors in spelling, punctuation, or capitalization, but meaning remains largely accessible. Errors are frequent enough to occasionally distract the reader. |
|  | 3–4 | Limited | Frequent errors in spelling, punctuation, and/or capitalization that interfere with readability. Patterns of error suggest limited command of English writing conventions. |
|  | 1–2 | Very Limited | Pervasive mechanical errors throughout the text. Spelling, punctuation, and capitalization are largely incorrect, severely impeding comprehension. |
